# Maize stomatal responses against the climate change

**DOI:** 10.3389/fpls.2022.952146

**Published:** 2022-09-20

**Authors:** Laura Serna

**Affiliations:** Facultad de Ciencias Ambientales y Bioquímica, Universidad de Castilla-La Mancha, Toledo, Spain

**Keywords:** climate change, drought, heat, maize, productivity, stomata

## Abstract

Drought and heat, in the context of climate change, are expected to increase in many agricultural areas across the globe. Among current abiotic stresses, they are the most limiting factors that influence crop growth and productivity. Maize is one of most widely produced crops of the world, being the first in grain production with a yield that exceeded 1.1 billion tons in 2021. Despite its wide distribution in semi-arid regions, it is highly vulnerable to climate change, which triggers important losses in its productivity. This article explores how maize yield may persevere through climate change by focusing on the stomatal regulation of gas exchange. The emerging picture unravels that maize copes with drought stress by reducing stomatal size and stomatal pore area, and increasing stomatal density, which, in turn, reduces transpiration and photosynthetic rate. When drought and heat co-occur, heat enhances stomatal response to drought stress. To avoid plant heat damage, the decline in stomatal aperture could trigger the expansion of the distance of action, from the longitudinal leaf veins, of ZmSHR1, which might act to positively regulate ZmSPCHs/ZmICE1 heterodimers, increasing the stomatal density. Only when drought is not very severe, elevated CO_2_ levels reduce yield losses. The knowledge of the upcoming climate changes together with the prediction of the developmental and physiological stomatal responses will allow not only to anticipate maize yield in the next years, but also to contribute to the correct decision-making in the management of this important crop.

## Introduction

Drought and heat stresses are the major limiting factors for crops growth and productivity (Fahad et al., 2017; Gupta et al., 2020; Jaldhani et al., 2022), and they cause the greatest annual loss of crops (Lipiec et al., 2013; Ray et al., 2015; Gupta et al., 2020). Worryingly, climate change, resulting from increasing emissions of greenhouse gases, is threatening crops yield, and food security, through increased temperatures and alterations of rainfall patterns (IPCC, 2021). The changes in rainfall patterns, which agree with climate models (Räisänen, 2002; Kharin et al., 2007; Wetherald, 2010; Fischer et al., 2013), are decreasing the frequency of the storms and increasing their intensity in many regions of the planet (Räisänen, 2002; Kharin et al., 2007; IPCC, 2013). This substitution of evenly distributed rainfall for an increased precipitation variability increases the risk of drought due to loss of water through runoff. Elevated temperatures also contribute to inducing drought because the rapid water loss from plant tissues and soil surface, and when they are too elevated can induce direct damage on crops (Wahid et al., 2007). Drought and heat stresses, both individually and in combination, have a deep impact on the agricultural sector, which, unfortunately, translates into a strong threat to food security.

**FIGURE 1:**
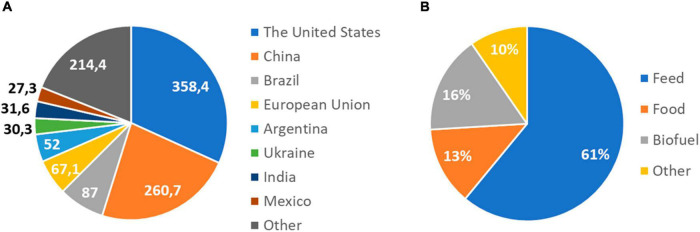
World maize production and uses. **(A)** Maize production by country in 2020/2021. The United States, China, and Brazil are the top producers. Production is expressed in million metric tons. Data source: USDA, Foreign Agricultural Service, Global Market Analysis. **(B)** Global uses of maize in 2021. Maize is used as food and animal feed, and as a source of biofuel. It can also be processed into a wide range of useful chemicals. Data source: OECD/FAO, 2021; forecast.

One of the world’s most widely produced crops is maize, being the first in grain production with a production that exceeded 1.1 billion tons in 2021 (FAS, USDA, 2022). It belongs to the grass family *Poaceae*, which includes more than 10,000 species (Kellogg, 1998), with other important crops such as wheat (*Triticum aestivum*), rice (*Oryza sativa*), or barley (*Hordeum vulgare*). Maize was domesticated from teosinte (*Zea mays* ssp. *Parviglumis*), at the tropical Balsas River valley in Mexico (Matsuoka et al., 2002; van Heerwaarden et al., 2011), and continued to spread north and south across the Americas (Matsuoka et al., 2002). Currently, it is cultivated across a wider area than any other major crop, being the United States, China, and Brazil the top producers (FAO, 2021; Figure 1A). Besides its primary use for food, it can also be processed into a variety of industrial products, including glue, industrial alcohol, and fuel ethanol (Ranum et al., 2014). Sixty one percent of global maize production is used as livestock feed and 13% for human consumption (OECD/FAO, 2021; Figure 1B). Despite the low percentage used directly for human consumption, it is an essential element of the diet of millions of people in Sub-Saharan Africa, where its use is expected to increase because the rapid growth of its population (OECD/FAO, 2021).

Despite its adaptation to a wide array of agro-ecologies, which explains its wide distribution, maize is highly vulnerable to climate change. It is cultivated in semi-arid environments, facing drought, heat, and combinations of these factors (Cairns et al., 2012; Zhao et al., 2016). Rojas et al. (2019), for example, by analyzing both the annual mean precipitation and specific growing seasons and areas found that reduced precipitation will impact, before 2040, maize production in southern Africa and Europe. Considering that the variability of precipitation is an essential factor because water availability during a given stage of plant developmental influences plant production at later stages of the life cycle (Brouwer and Heibloem, 1986; Kranz et al., 2008; Al-Kaisi and Broner, 2009; Hashim et al., 2012; Halubok and Yang, 2020), it is likely that more regions of the globe with maize crops will be concerned. In addition, in the context of climate change, not only changes in rainfall patterns affect maize growth, but also increases in temperature. Certainly, it is known that temperatures above 35°C negatively impact the vegetative and reproductive growth of maize, from germination to grain filling (Hatfield et al., 2011). The Russian invasion of Ukraine will also affect maize production, with a decline of Ukraine maize production for 2022/2023 of 54% relative to last year (FAS, USDA, 2022). Moreover, the impact of climate change on maize will be more pronounced considering that global population is predicted to rise, in the least drastic scenario, from 7.7 billion currently to 9,7 billion in 2050 (Adam, 2021). The greatest climatic impact, in addition, falls on South Africa (SADC, 2016; Rojas et al., 2019), where maize is an essential element of the diet of its population (SADC, 2016; OECD/FAO, 2021), and where, together with other regions in Southern Africa, the persistent socioeconomic vulnerability enhances the negative impact of climate change (Leichenko and O’Brien, 2002; Niang et al., 2015).

This article explores the consequences of climate change on both maize productivity and stomatal development (stomatal density and stomatal size) and function. Drought, through alterations in the stomatal development and function, reduces both transpiration and photosynthetic rate (Zhao et al., 2015; Hussain et al., 2019). When drought and heat co-occur, plants experience a reduction in transpiration rate, photosynthetic rate, and biomass accumulation, which are more severe than those induced by drought stress individually (Hussain et al., 2019). In addition, the alterations in stomatal function are physiologically buffered, to avoid plant heat damage, with changes in the stomatal density possibly induced by the expansion of the distance of action, from the longitudinal leaf veins, of ZmSHR1. Given that crop yields must improve despite the potentially negative consequences of increasing temperatures and changing precipitation patterns, this stomatal response to climate change alerts about the future of maize cultivation, and it demands a search for solutions to deal with the impact of climate change on its productivity. Even more so considering that only when drought is not very severe, maize benefits from increased CO_2_ levels reducing yield losses (Webber et al., 2018). This mitigation of the drastic effects of drought is due to a reduction in stomatal transpiration, which improves water use efficiency, and consequently, the water content of the soil (Long et al., 2004; Leakey et al., 2006, 2009; Ghannoum, 2009; Manderscheid et al., 2014).

## Effects of drought and heat on maize crop productivity

Although there are other factors that affect maize production, drought and heat are, without a doubt, two of the most important. Certainly, there are several works that show the relevance of these factors on maize yield in distinct parts of the world (Table 1). Maitah et al. (2021), for example, demonstrated the influence of precipitation, from 2002 to 2019, on maize yield in the Czechia. They found that both total yield and yield rate increased from 1961 to 2010, but they dropped after 2010, just when precipitation also decreased. After 2010, there was also a trend of increasing temperature that correlates with a decrease in total yield and yield rate (Maitah et al., 2021). Data from more than 20,000 historical maize trials in Africa, from 1999 to 2007, combined with daily temperature and precipitation data, showed that each additional degree day above 30°C reduced the final yield by 1% under optimal rainfed conditions, and by 1.7% under drought ones (Lobell et al., 2011). This is telling us that the ability of maize to cope with rising temperatures depends on the availability of water. Certainly, plant cooling takes place through transpiration (Curtis, 1936), which needs soil moisture. Outside Europe and Africa, specifically in Khyber Pakhtunkhwa (Pakistan), an analysis of maize yield between years 1996 and 2015 also showed that precipitation has a positive effect in maize productivity, while elevated temperatures have a deleterious impact (Khan et al., 2019). In the United States, maize yield losses, from 1959 to 2004, were due to increased evaporative demand and subsequent water supply depletion, which was induced by high temperatures (Lobell et al., 2013). Together, this suggests that, at least up to a certain temperature, drought, induced by a deficiency of precipitation or elevated temperatures, causes a decrease in maize productivity.

**TABLE 1:** Observations and estimations of maize yield and drivers of its change.

Region	Period	Drivers of yield changes and effect on yield	References
Czechia	2002–2019	Decrease in precipitation and increase in temperature decreased from 7.73 t/ha (2001–2010) to 7.67 (2011–2019) maize yield, even considering technological and management improvement in production	Maitah et al., 2021
Africa	1999–2007	Each additional degree day spend above 30°C, changed the final yield by −1% under optimal rainfed conditions, and by −1.7% under drought ones	Lobell et al., 2011
Khyber Pakhtunkhwa	1996–2015	Increase in precipitation increased maize yield, and increase in temperature decreased maize yield	Khan et al., 2019
The United States	1959–2004	Increase in evaporative demand induced by elevated temperatures decreased maize yield	Lobell et al., 2013
Europe	2050	Drought will change maize yield −20%	Webber et al., 2018
Turkey	2050	Drought and heat will change maize yield −10.1%	Dellal et al., 2011
Sub-Saharan Africa	2056–2065 and 2081–2090	Drought or heat, depending on space, will change maize yield from >+6 to <−33%	Waha et al., 2013
The United States	2050	Drought or heat, depending on the climate scenario, will change maize yield from −39 to −68% (relative to 2013–2017). And from −13 to +62% (relative to 2013–2017), incorporating to the model the estimated effects of climate-neutral technological advances	Yu et al., 2021
World	End-of-century	Climate change will change maize yield from +5 to −6% (SSP126) and from +1 to −24% (SSP585), excluding changing farming practices and maize adaptations	Jägermeyr et al., 2021

Estimations of the effect of drought and heat on maize yield in various regions of the world, from mid- to late-21st century, have been also realized (Table 1). In Europe, climate change by 2050 will reduce maize yield by 20% (Webber et al., 2018). In addition, drought stress versus heat stress is the main driver of losses for maize yield, even in low-yielding years (Webber et al., 2018). In agreement with modeling analysis (Schauberger et al., 2017), elevated CO_2_ concentration will be able to mitigate such losses only when drought is not too severe (Webber et al., 2018). It is also expected a drop of 10.1% in maize yield toward the middle of the century in Turkey, and it is associated to drought and/or heat stress (Dellal et al., 2011). In sub-Saharan Africa, maize yield was estimated for two 10-year periods, 2056–2065 and 2081–2090, unraveling changes from >+6 to <−33% (Waha et al., 2013). The authors found that the importance of changes in temperature and precipitation in maize yield will depend on the study region. For example, in southern parts of Mozambique and Zambia, the Sahel and parts of eastern Africa, a reduction of the wet season precipitation will cause a decrease in maize yield, prevailing over the effect of increased temperatures (Waha et al., 2013). Although, as the authors suggested, the model may have underestimated the damage that elevated temperatures will produce. Projections of changes in precipitation and temperature in the United States showed that maize yield, by 2050 and relative to 2013–2017 period, will reduce by 39–68% depending on the climate scenario (Yu et al., 2021). When the authors incorporated to the model the estimated effects of climate-neutral technological advances, the net change in yield ranged from (−)13 to 62%, questioning, interestingly, the usefulness of scientific efforts in adapting crops to extreme conditions of heat and drought (Yu et al., 2021). Considering the total maize production in the world, twenty-first-century projections using state-of-the-art climate and crop model suites, but excluding changing farming practices, and adaptations such as breeding hardier crop varieties, suggest that mean maize productivity, at the end-of-century, will shift from +5 to −6% (SSP126) and from +1 to −24% (SSP585) (Jägermeyr et al., 2021).

Despite some models omit CO_2_ fertilization effect (Dellal et al., 2011; Waha et al., 2013), which alleviates yield losses when drought is not too intense (Webber et al., 2018), drought and heat are reducing, and will continue to do so, maize yield in many regions of the world. The intensity of this effect depends not only on the genotypes, but also on environmental conditions and, therefore, on time and location of these crops. The inclusion in the models of changing farming practices, adaptations such as breeding hardier crop varieties and economic incentives is essential to anticipate the effect of climate change on maize crop yield and to design strategies for its mitigation. Because in the next 50 years climate extreme events will alternate with normal ones (IPCC, 2021), and varieties adapted only to extreme events reduce their yield (Yu et al., 2021), there is an urgent need not to make varieties more resilient to extreme drought and heat, but to adapt these varieties to a wide variety of conditions.

## Maize stomatal response to drought and heat stresses

Plants have developed multiple responses at the developmental, physiological, and molecular levels that enable them to escape, avoid, and/or tolerate unfavorable environmental conditions (Gupta et al., 2020; Chávez-Arias et al., 2021). Avoidance of drought and/or heat stress damage includes changes in stomatal number and/or function (Gupta et al., 2020; Chávez-Arias et al., 2021). Stomatal pores open to absorb CO_2_ for photosynthesis, and close to prevent water loss through transpiration (Blatt et al., 2017). It is widely known that drought stress induces stomatal closure reducing water loss (Taiz and Zeiger, 2006). However, in some regions of the world, maize not only faces low water availability, but also elevated temperatures (Hu et al., 2015; Zhao et al., 2016). For a century, it has been known that transpiration reduces leaf temperature (Curtis, 1936). Therefore, stomatal closure to prevent transpiration, also triggers leaf heating. But how does maize solve the dilemma of avoiding water loss and, at the same time, heating the leaves when growing under both drought and high temperatures?

**FIGURE 2:**
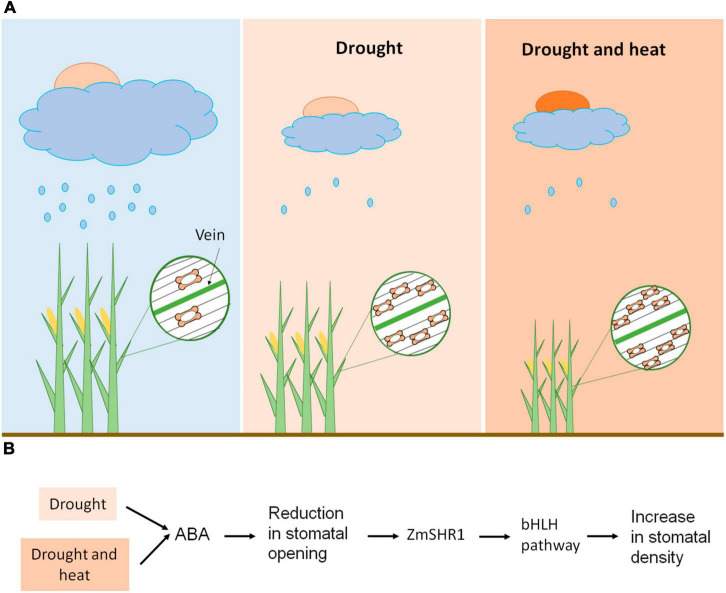
Maize stomatal response to climate change. **(A)** Heat enhances the maize stomatal responses to drought stress. Drought reduces stomatal size and opening, and increases stomatal density, which, in turn, reduces transpiration and photosynthetic rate. When drought and heat coexist, plants experience a reduction in transpiration rate and photosynthetic rate, possibly related to a reduction in stomatal size and opening, accompanied by an increase in stomatal density. This triggers a reduction in biomass accumulation, which is more severe than that induced by drought stress. **(B)** Possible molecular mechanisms of stomatal response to climate change. Drought stress, individually or in combination with high temperatures, reduces stomatal opening by increasing abscisic acid (ABA) levels. This stomatal response could trigger the increase of the distance of action of ZmSHR1 from the longitudinal leaf veins. ZmSHR1 might act to positively regulate ZmSPCHs/ZmICE1 heterodimers, increasing the number of stomatal files and, consequently, the stomatal density.

Specifically in maize, with the typical grass stomata consisting of two dumbbell-shaped guard cells (Stebbins and Shah, 1960; Serna, 2011), severe water deficit (40–50% field capacity) leads to a decrease in the size and opening of the stomata and an increase in stomatal density (Zhao et al., 2015; Figure 2A). The latter is possibly associated with the need for cooling through transpiration. Anyway, this stomatal response to drought negatively impacts stomatal conductance, photosynthetic rate and transpiration (Zhao et al., 2015; Hussain et al., 2019). The reduction in the stomatal size has an important advantage because it increases the speed of stomatal movement (Aasamaa et al., 2001; Hetherington and Woodward, 2003), resulting in a decrease in water loss by transpiration. But it also implies a reduction in the assimilation of photosynthetic CO_2_, and in the yield of the plant. Nonetheless, the negative correlation between stomatal density and transpiration rate in maize is stronger than that with photosynthetic rate, indicating that leaf water use efficiency tends to increase (Zhao et al., 2015). As expected, when the temperature increases, the stomatal aperture area does too, which increases the stomatal conductance and the rate of transpiration (Zheng et al., 2013), avoiding the heating of the leaves. Hussain et al. (2019) also found that heat stress (38°C for 15 days) increases the rate of transpiration, but it decreases the photosynthetic rate (these changes were not statistically significant). This decrease in photosynthetic rate is obviously due to non-stomatal limitations, such as alterations in electron transport capacity and activity (Way and Oren, 2010; Zafar et al., 2018). Certainly, exceeding 35°C degrades maize chlorophyll (Hatfield et al., 2011; Hussain et al., 2019), and compromises protein activity with strong impact on carbon assimilation (Chaves et al., 2016). However, the combination of heat and drought generates a reduction in stomatal conductance, transpiration rate, photosynthetic rate, biomass accumulation and, ultimately, yield, with these reductions being more severe than those induced only under drought stress (Hussain et al., 2019). Therefore, heat enhances the stomatal response to drought, possibly associated with a reduction in stomatal pore area accompanied by an increase in stomatal density, which in turn reduces transpiration, but it also increases leaf temperature (Figure 2A). This reduction in transpiration associated with high water use efficiency has costs in terms of lower rates of CO_2_ assimilation and reduced yield, possibly through a direct decrease in CO_2_ uptake, and an increase in leaf temperature that negatively impacts protein activity. Taking theses stomatal responses into account, it is likely that the increased frequency of extreme events induced by climate change, such as heat waves, will exacerbate maize yield loss.

## Only under certain drought conditions, maize benefits from elevated CO_2_ levels

Climate change includes not only rising temperatures, changes in precipitation patterns and increasing frequency of extreme weather events, but also increased atmospheric concentrations of CO_2_. In maize, heat enhances the stomatal response to drought, decreasing water loss through transpiration (Hussain et al., 2019). This protective response to drought has costs in terms of lower CO_2_ assimilation, which is manifested by a decrease in the photosynthetic rate and, by extension, in the accumulation of biomass (Hussain et al., 2019). Will maize benefit from increased CO_2_ levels, avoiding a decline in growth and yield, when water is scarce, and temperatures rise?

Increased atmospheric concentrations of CO_2_ stimulate photosynthesis and yield of C3 species (Long et al., 2004; Leakey et al., 2009; Kimball, 2016). However, C4 species concentrate CO_2_ at the site of Rubisco, and the enzyme is saturated with the current CO_2_ levels (Furbank et al., 1989; Jenkins et al., 1989; von Caemmerer and Furbank, 2003; Ghannoum, 2009). Therefore, the increase of CO_2_ concentration should not induce any effect on the photosynthetic rate of these plant species. Agree with this, several works have shown the insensitivity of C4 species, including maize (Leakey et al., 2006; Markelz et al., 2011; Manderscheid et al., 2014; Ruiz-Vera et al., 2015), to increases in CO_2_ levels under sufficient water supply (Leakey et al., 2009; Kimball, 2016), and except for one paper showing that maize benefits from CO_2_ enrichment (Driscoll et al., 2006). However, while the effects of increased temperature on photosynthesis and growth of well-watered maize plants remain unchanged at elevated CO_2_ levels compared to current ones (Kim et al., 2007), when water becomes limiting, increased levels of atmospheric CO_2_ levels improve their photosynthesis and growth (Leakey et al., 2006; Markelz et al., 2011; Manderscheid et al., 2014). However, and according to model analysis (Schauberger et al., 2017), CO_2_ levels can alleviate the negative impact of drought only when it is not too severe (Webber et al., 2018).

In C4 species, the alleviation of the negative effects of drought under elevated CO_2_ levels is due to a reduction in stomatal transpiration, improving water use efficiency and, consequently, the water content of the soil (Long et al., 2004; Leakey et al., 2006, 2009; Ghannoum, 2009; Manderscheid et al., 2014). However, this reduction in transpiration, on the other hand, increases leaf temperature (Curtis, 1936; Kimball et al., 1999; Gray et al., 2016), which may intensify heat stress, impacting maize yield (Ruiz-Vera et al., 2015). Therefore, maize may benefit from increased CO_2_ levels only when the drought is not too severe, and temperatures do not reach very extreme values.

## Possible molecular mechanism of maize stomatal development in response to climate change

Changes in stomatal density can greatly impact a plant’s water use efficiency and, consequently, drought tolerance. Grasses develop their stomata in rows positioned at the flanks of underlying longitudinal leaf veins (Stebbins and Shah, 1960). This position of stomatal files may result from an inhibitory signal transmitted from the vein to overlying epidermal cells and/or from an inductive signal transmitted to epidermal cells at a specific distance from the vein (Hernandez et al., 1999). One such candidate for this inductive signal is *ZmSHR1*, since transgenic rice lines expressing this gene in an expanded domain, compared to the vascular-specific expression domain of its orthologous *OsSHR2* gene, produce supernumerary stomatal files between veins (Schuler et al., 2018).

In *Arabidopsis*, entry into stomatal lineage is controlled by the basic helix-loop-helix (bHLH) protein SPEECHLESS (AtSPCH) and its more distantly related bHLH heterodimer partners INDUCER OF CBF EXPRESSION1 (AtICE1) and SCREAM2 (AtSCRM2) (MacAlister et al., 2007; Kanaoka et al., 2008). Proteins encoded by the duplicated *SPCH* homologs in *Brachypodium*, BdSPCH1 and BdSPCH2, also redundantly control stomatal lineage initiation, with loss-of-function of both *BdSPCH1/2* (*bdspch1 bdspch2*) triggering a stomata-less phenotype, and gain-of-function by overexpression of *BdSPCH2* inducing ectopic stomatal development in new cell files (Raissig et al., 2016). In addition, *BdICE1*, but not *BdSCRM2*, drives stomatal lineage initiation (Raissig et al., 2016). This suggests that BdSPCHs/BdICE1 heterodimers regulate entry into the stomatal lineage. In maize, there are three copies of *SPCH-*like genes and one copy of *ICE1/AtSCRM2*-like genes (McKown and Bergmann, 2020), suggesting that stomatal initiation is also controlled by ZmSPCHs/ZmICE1 heterodimers.

ZmSHR1, through an unknown mechanism, might act to positively regulate these ZmSPCHs/ZmICE1 heterodimers in epidermal files that flank leaf veins and, thus, to promote stomatal initiation (Figure 2B). Thus, drought stress, by increasing abscisic acid (ABA) levels, reduces stomatal opening (Munemasa et al., 2015; Zhao et al., 2015), which, to avoid plant heat damage, could increase the number of stomatal files and, consequently, the stomatal density, by expanding the expression domain of *ZmSHR1* (Figure 2B). Drought could also decrease the stomatal distance within epidermal files, but the molecular mechanism behind this regulation is unknown. Under adequate water supply, *ZmSHR1* expression in expanded domains by genetic manipulation would produce supernumerary stomatal rows between veins and, consequently, increased stomatal density. This would reduce excess heat by increasing transpiration and would, possibly, improve maize yield.

## Future considerations

Climate change is increasing the frequency of extreme events such as heat waves (IPCC, 2021). The heat is intensifying the effect of the drought in maize by decreasing gaseous exchange through the production of smaller stomata and reducing their opening (Zhao et al., 2015; Hussain et al., 2019). Since photosynthesis is saturated at current CO_2_ levels in C4 species (Kimball, 2016), this response could be beneficial in mitigating hydraulic demand. However, decreased transpiration will increase leaf temperature, which will cause damage to plants in specific locations of the planet. Under this climatic context, the combination of plant breeding and agronomic management is required to avoid yield losses. Under adequate water supply, targeted genetic manipulation through the production of genotypes characterized by an increase in stomatal size and/or density could regulate leaf temperature by increasing transpiration, thus preventing tissue damage. However, under water restrictions, close monitoring of plant temperature could prevent tissue damage caused by, for example, heat waves, through timely irrigation. Earlier sowing could also help rainfed maize adapt to climate change in some regions with higher water demand in warmer periods. Even more if we consider that the greatest yield losses occur when drought stress prevails in the pre-tasseling stage (Anjum et al., 2017), and high temperatures near the anthesis stage (Gabaldón-Leal et al., 2016). In any case, adaptation strategies must be local, and they must consider both agronomic management and well-adapted genotypes.

Since varieties adapted only to extreme drought and/or heat reduce their yield (Yu et al., 2021), selection or production of varieties more resilient to extreme events, but also adapted to a wide variety of conditions, is essential to avoid yield losses. To achieve this, genetic modifications aimed at modifying stomatal density and/or opening could be combined with those aimed at modifying enzymes that regulate the photosynthetic process. For example, as Pignon and Long (2020) suggested, maize mutants with reduced carbonic anhydrase activity could be combined with transgenic maize overexpressing Rubisco to improve photosynthesis and water use efficiency under elevated CO_2_ levels. In addition, the combination of modifications in these characters with others that are not related to the stomatal response could improve the adaptation of maize to climate change.

## Author contributions

The author confirms being the sole contributor of this work and has approved it for publication.
